# Phytic acid-modified manganese dioxide nanoparticles oligomer for magnetic resonance imaging and targeting therapy of osteosarcoma

**DOI:** 10.1080/10717544.2023.2181743

**Published:** 2023-03-01

**Authors:** Qian Ju, Rong Huang, Ruimin Hu, Junjie Fan, Dinglin Zhang, Jun Ding, Rong Li

**Affiliations:** a College of Chemistry, Chongqing Normal University, Chongqing, China; b Department of Chemistry, College of Basic Medicine, Army Medical University (Third Military Medical University), Chongqing, China; c Department of Urology, Southwest Hospital, Army Medical University (Third Military Medical University), Chongqing, China; d Department of Clinical Laboratory, the 958th Hospital of Chinese People’s Liberation Army, Chongqing, China; e Department of Ultrasonics, Southwest Hospital, Army Medical University (Third Military Medical University), Chongqing, China

**Keywords:** Phytic acid, manganese dioxide nanoparticles, osteosarcoma, magnetic resonance imaging, targeting therapy

## Abstract

Osteosarcoma is the most common malignant tumor in the skeletal system with high mortality. Phytic acid (PA) is a natural compound extracted from plant seeds, which shows certain antitumor activity and good bone targeting ability. To develop a novel theranostics for magnetic resonance imaging (MRI) and targeting therapy of osteosarcoma, we employed PA to modify manganese dioxide nanoparticles (MnO_2_@PA NPs) for osteosarcoma treatment. The MnO_2_ NPs oligomer was formed by PA modification with uniformed size distribution and negative zeta potential. Fourier-transform infrared spectroscopy, X-ray diffraction, energy dispersive spectroscopy, X-ray photoelectron spectroscopy, and thermogravimetric analysis demonstrated that PA has been successfully modified on MnO_2_ NPs, and the structure of MnO_2_@PA NPs is amorphous. *In vitro* experiments demonstrated that MnO_2_@PA NPs oligomer can be efficiently internalized by tumor cell, and the internalized NPs can react with H_2_O_2_ under acid microenvironment to produce Mn^2+^ and O_2_. *In vivo* experiments demonstrated that MnO_2_@PA NPs oligomer can passively accumulate in tumor tissue, and the accumulated NPs can produce Mn^2+^ and O_2_ for MRI and targeting therapy of osteosarcoma. In conclusion, we prepared a novel bone-targeting nano theranostics for MRI and therapy of osteosarcoma.

## 1. Introduction

Osteosarcoma is the most common malignant tumor in the skeletal system, accounting for 20%–34% of the primary malignant bone tumor with extreme invasiveness and metastasis as well as dismal prognosis (Rathore & Van Tine, 2021). Currently, surgery was the preponderant clinical treatment, but only patients with early-stage osteosarcoma could be cured via surgical resection. In addition, the five-year survival rate after surgery was less than 70% (Siegel et al., 2021). Surgery combined with neoadjuvant chemotherapy can significantly increase the survival rate of patients. Classical anticancer drugs such as cisplatin, doxorubicin, and methotrexate have shown good therapeutic effects to osteosarcoma (Jiang et al., 2022). However, chemical drugs are likely to cause numerous side effects and bone marrow microenvironment-associated drug resistance is inevitable (Yang & Tian et al., 2020).

In recent years, various nanoformulations such as inorganic nanoparticles (NPs) (Li et al., 2020), liposomes (Jing et al., 2022), nanogel (Zhang et al., 2018), lipid NPs (Peira et al., 2022), and polymeric NPs (Heyder et al., 2021) had been widely exploited for targeting treatment of osteosarcoma (Ambrosio et al., 2021; Wu et al., 2022). Nanoformulations can target and deliver therapeutics to osteosarcoma region to improve therapeutic benefits and decrease adverse effects of therapeutics (Jo et al., 2015). Otherwise, NPs can alter tumor microenvironment to decrease tumor cells survival through a serial of chemical, physical, and photogenic reactions. For example, manganese dioxide (MnO_2_) NPs synthesized by chemical (Lim et al., 2021) or biological methods (Liu et al., 2021) can catalyze the conversion of endogenous hydrogen peroxide (H_2_O_2_) in tumor region into O_2_ due to its catalase-like activity, thereby alleviating tumor hypoxia. The O_2_ generated by MnO_2_ can facilitate the cancer cell cycle status to S phase, which is sensitive to chemotherapeutic drugs (Guo et al., 2020). In addition, MnO_2_ NPs can enhance radiotherapy efficacy of tumors (Liu et al., 2020; Yang & Ren et al., 2020). MnO_2_ NPs also play an important role in osteosarcoma treatment. For example, alendronate/K7M2 cell membranes-coated hollow MnO_2_ NPs encapsulated ginsenoside Rh2 was employed for immuno-chemodynamic combination therapy of osteosarcoma (Fu et al., 2022). In addition to chemodynamic therapy, the Mn^2+^ ions produced by MnO_2_ NPs can be used as a magnetic resonance imaging (MRI) contrast, which displayed good biodegradation and biocompatibility *in vivo* (Yang et al., 2018). Consequently, MnO_2_ NPs can potentially serve as a theranostics for T1-weighted MRI and therapy of osteosarcoma or other tumor. However, how to improve the targeting capacity of MnO_2_ NPs to osteosarcoma need to be further investigated.

Aptamers (Niu et al., 2022), diphosphonate (Wu & Wan, 2012), and aspartic acid-related oligopeptides (Ogawa et al., 2017) exhibited good targeting ability to bones. However, their applications were restricted due to sophisticated synthetic methods or unpredictable biosafety. Phytic acid (PA), an organic phosphoric acid compound extracted from plant seeds, has been widely used as food additives (Zhou et al., 2019), antioxidant (Lux et al., 2022), preserving agent (Zhao et al., 2022), and chelating agent (Chen et al., 2018). PA has good biocompatibility since it was detected in mammalian cells. Interestingly, PA showed certain antitumor activities on colon tumor (Vucenik et al., 2020). PA also displayed special bone-targeting capability due to its strong chelating ability to calcium ion of bone. Consequently, PA-modified NPs was employed for targeting treatment of bone tumors (Zhou & Fan et al., 2019; Wang et al., 2020).

Herein, we hypothesized that PA-modified MnO_2_ NPs (MnO_2_@PA NPs) could improve the targeting capability and therapy efficacy of MnO_2_ for osteosarcoma treatment. To verify this hypothesis, we fabricated MnO_2_@PA NPs and investigated the *in vivo* targeting ability and therapeutic efficacy of NPs for osteosarcoma on a mice model. Fourier-transform infrared spectroscopy (FTIR), X-ray diffraction (XRD), energy dispersive spectroscopy (EDS), X-ray photoelectron spectroscopy (XPS), and thermogravimetric analysis (TGA) indicated that PA was successfully modified on MnO_2_ NPs, and the fabricated MnO_2_@PA NPs was amorphous. Transmission electron microscopy (TEM) results confirmed that a MnO_2_@PA NPs oligomer was formed by PA modification. *In vitro* experiments demonstrated that MnO_2_@PA NPs could be efficiently internalized by various tumor cells and the internalized NPs can release Mn^2+^ under acidic microenvironment. *In vivo* experiments verified that MnO_2_@PA NPs could enhance T1 weighted MRI of tumor and significantly suppress tumor growth in a 143B tumor-bearing mice model. The theranostics exhibits wide prospect clinical applications for MRI and therapy of osteosarcoma.

## 2. Materials and methods

### 2.1. Regents and cells

Potassium permanganate (KMnO_4_) and manganese sulfate (MnSO_4_) were obtained from Sinopharm Chemical Reagent Co., Ltd. (Shanghai, China). PA, *W* = 70% was provided from Sigma-Aldrich (St. Louis, USA). Fetal bovine serum (FBS), Dulbecco’s modified Eagle’s medium (DMEM) solutions, and Roswell Park Memorial Institute 1640 (RPMI 1640) solution were purchased from Thermo Fisher Scientific (Waltham, MA, USA). Cell Counting Kit-8 (CCK-8, 480 T) and dichlorofluorescein diacetate (DCFA-DA, 2.5 μm) were received from Beyotime Biotechnology Co., Ltd. (Shanghai, China). H_2_O_2_ colorimetric assay kit (480T) was obtained from Abbkine Biotechnology Co., Ltd. (Shanghai, China). BBcellProbe® P01 probe was purchased from Bestbio Co., Ltd. (Shanghai, China).

All cells including RAW 264.7 (mouse monocyte macrophage), U2OS, 4T1, Ishikawa, MC 38, Panc 02, SW480, and 143B cells were obtained from American type culture collection (ATCC; Manassas, VA, USA). These cells were cultured in DMEM media with 10% FBS, 100 µg/mL streptomycin, and 100 µg/mL penicillin in a 37 °C incubator with fractional concentration of 5% carbon dioxide.

### 2.2. Synthesis of MnO_2_ NPs and MnO_2_@PA NPs

A reported method was employed to synthesize MnO_2_ NPs (Marin et al., 2020). In brief, 2.455 g of MnSO_4_•H_2_O and 2.224 g of KMnO_4_ were dissolved in 50 mL of ultrapure water and heated to 80 °C, respectively. About 100 mL of distilled water was added to a three-necked flask, then the heated MnSO_4_ and KMnO_4_ solution were slowly added to the three-necked flask with 1000 rpm stirring. A black suspension was obtained after 2 h reaction at 80 °C. The suspension was cooled to room temperature, ultrasonically dispersed for 10 min, filtered with suction, rinsed three times with distilled water, and dried in a drying oven at 80 °C for 12 h to obtain MnO_2_ NPs.

To prepare MnO_2_@PA NPs, 0.0512 g of KMnO_4_ and 0.25 mL of PA were dissolved in 20 mL deionized water and reacted at 50 °C for 8 h. After finished the reaction, the solution was further stirred at room temperature for 24 h, then centrifuged at 6000 rpm for 5 min and washed three times with deionized water to obtain MnO_2_@PA NPs. The reaction conditions were optimized with the same method.

### 2.3. Characterization of the MnO_2_@PA NPs

The size, polydispersity index (PDI), and zeta potential of MnO_2_@PA NPs were measured by dynamic light scattering (DLS) and laser Doppler anemometry with a Malvern Zetasizer (Nano ZS, Malvern, UK). To check the stability of the NPs, 0.5 mg/mL of MnO_2_@PA NPs was used to determine the size and PDI of NPs at 0, 7, 14, and 28 days.

The structure of MnO_2_@PA NPs was characterized by FTIR (FTIR-8400S, Shimadzu, Japan). The morphology of the NPs was observed by TEM (JEM-2100F, JEOL, Japan). The phase structure of the NPs was analyzed by X-ray diffractometer (Max-2550, Rigaku Corporation, Japan). The elements and valence states of manganese and phosphorus in NPs were analyzed by XPS (Escalab 250Xi spectrometer, Thermo Fisher Scientific, USA). The element species and contents of NPs were determined by EDS (Elite T, EDAX Inc, USA). The thermal stability of NPs was tested by thermogravimetric/differential scanning calorimeter (TG209F3, NETZSCH, Germany).

### 2.4. Cytotoxicity assay

RAW 264.7, U2OS, 4T1, Ishikawa, MC 38, Panc 02, 143B, and SW480 cells were cultured in 96-well plates (10^4^ cells/well) and incubated overnight. To evaluate the cytotoxicity of PA and MnO_2_@PA NPs, cells were incubated with different concentrations of PA and MnO_2_@PA NPs (0, 12.5, 25, 50, 75, 100, 125, 150 μg/mL) for 4 or 24 h. After incubation, cells were washed with phosphate-buffered saline (PBS) to remove PA and MnO_2_@PA NPs, and 100 μL medium containing 10% CCK-8 solution was added to each well with another 30-min incubation, followed by optical density was measured at 450 nm using a Thermo Multiskan Spectrum spectrophotometer (Varioskan Flash, Thermo Scientific Inc., USA).

### 2.5. Detection of pH value, ROS, and H_2_O_2_ in cells

Intracellular reactive oxygen species (ROS) levels were detected by DCFH-DA. To evaluate the varieties of ROS in cells with MnO_2_@PA NPs treatment, cells were co-cultured with MnO_2_@PA NPs in six-well plates (10^6^ cells/well) for 4 h, then the medium was discarded and the cells were washed with PBS to remove MnO_2_@PA NPs. After that, trypsinization was added and the cell suspension was transferred into a 1.5-mL Eppendorf tube for centrifugation (1000 rpm, 5 min). The centrifuged cells were re-suspended in 1 mL of serum-free medium, and 1 μL of DCFH-DA reagent was added to each well with 20 min incubation at 37 °C. The cells were then washed with PBS, re-suspended in serum-free medium, and analyzed on a flow cytometer (NovoCyte. ACEA, USA).

To determine the intra- and extracellular H_2_O_2_ concentration varieties, MnO_2_@PA NPs were incubated with cells for 4 h, and the intra- and extracellular H_2_O_2_ concentrations were determined by H_2_O_2_ kit according to manufacturer’s instructions.

BBcellProbe® P01 probe was used to detect the pH value in tumor cells. Cells were incubated with PA, MnO_2_ NPs, MnO_2_@PA NPs for 4 h to determine pH value viability in cells. After incubation, the medium was discarded and cells were washed with Hank’s Balanced Salt Solution (HBSS) to remove PA, MnO_2_ NPs, or MnO_2_@PA NPs. After washing, trypsin digestion was added and the cell suspension was centrifuged (1000 rpm, 5 min). The centrifuged cells were washed with HBSS again and re-suspended in 1 mL of HBSS. Except for the blank control group, 1 μL of BBcellProbe® P01 probe was added to each well and incubated at 37 °C for 30 min. After incubation, cells were washed with HBSS and re-suspended with 1 mL of HBSS for analysis on a flow cytometer.

### 2.6. Cell uptake of NPs

To evaluate the cellular uptake behavior of MnO_2_@PA NPs, cells were incubated with MnO_2_@PA NPs for 24 h. After incubation, cells were digested with trypsin and centrifuged at 1000 rpm for 5 min. The collected cells were washed and centrifuged. The centrifuged cells were re-suspended in 2 mL of DMEM medium and the number of cells were counted by inductively coupled plasma mass spectrometry (ICP-MS) (Agilent ICP-MS 7800, Agilent, USA) to determine the contents of manganese in the cells.

### 2.7. Preliminary evaluation of biosafety of MnO_2_@PA NPs

To evaluate bio-safety of MnO_2_@PA NPs *in vivo*, 20 female Kunming mice were divided into four groups, in which the control group was received 100 μL of saline, and other groups were received 10, 20, and 40 mg/kg of MnO_2_@PA NPs, respectively. Drug administration was performed every four days, three times in total. Mice were monitored and weighed every other day. After two weeks, mice were sacrificed and blood was collected for hematological analysis. The main organs of the mice including heart, liver, spleen, lung, and kidney were collected and fixed with 4% paraformaldehyde for hematoxylin–eosin (H&E) staining.

### 2.8. In vivo antitumor evaluation

Four-week-old female BALB/c nude mice were obtained from the Experimental Animal Center of Army Medical University (Chongqing, China) and kept in a SPF-level sterile animal room. A total of 1 × 10^6^ 143B cells re-suspended in sterile RPMI 1640 medium were subcutaneously implanted into the right back of mice to establish a bone tumor model. The tumor volume was measured and calculated as follows:

$$V\left({\text{mm}}^{3}\right)=L\times W^{2}/2$$

*V* is the tumor volume of nude mice; *L* is the longest diameter of the tumor; and *W* is the shortest diameter of the tumor. When the tumor volume grew to 100 mm^3^, mice were randomly divided into four groups (*n* = 5). Control group received 100 mL of PBS. Treatment groups received 20 mg/kg of PA, MnO_2_ NPs, or MnO_2_@PA NPs per mice, respectively. Drug administration was performed every four days, three times in total. Tumor volumes and mice weights were measured every two days. After treatment, mice were sacrificed, and the resected tumors were weighted and immersed in 4% paraformaldehyde solution for histological examination and immunohistochemical analysis. The manganese contents in tumor tissues were detected by ICP-MS.

### 2.9. In vivo T1-weighted MRI

When tumor volume reached 200 mm^3^, mice received 20 mg/kg of MnO_2_@PA NPs via tail vein injection. T1-weighted MRI was performed at 0, 0.5, 2, 4, 8, 24, and 48 h after administration.

### 2.10. Statistical analysis

Results were expressed as mean ± standard deviation (SD). All measurements included at least three independent experiments. One-way variance (ANOVA) was used for data analysis. Tukey’s multiple comparison test was used for more than three groups, and Student’s *t* test was used for two groups. Statistical significance was defined as *p < .05, **p < .01, and ***p < .01.

## 3. Result and discussion

### 3.1. Preparation and characterization of NPs

**Figure 1.**
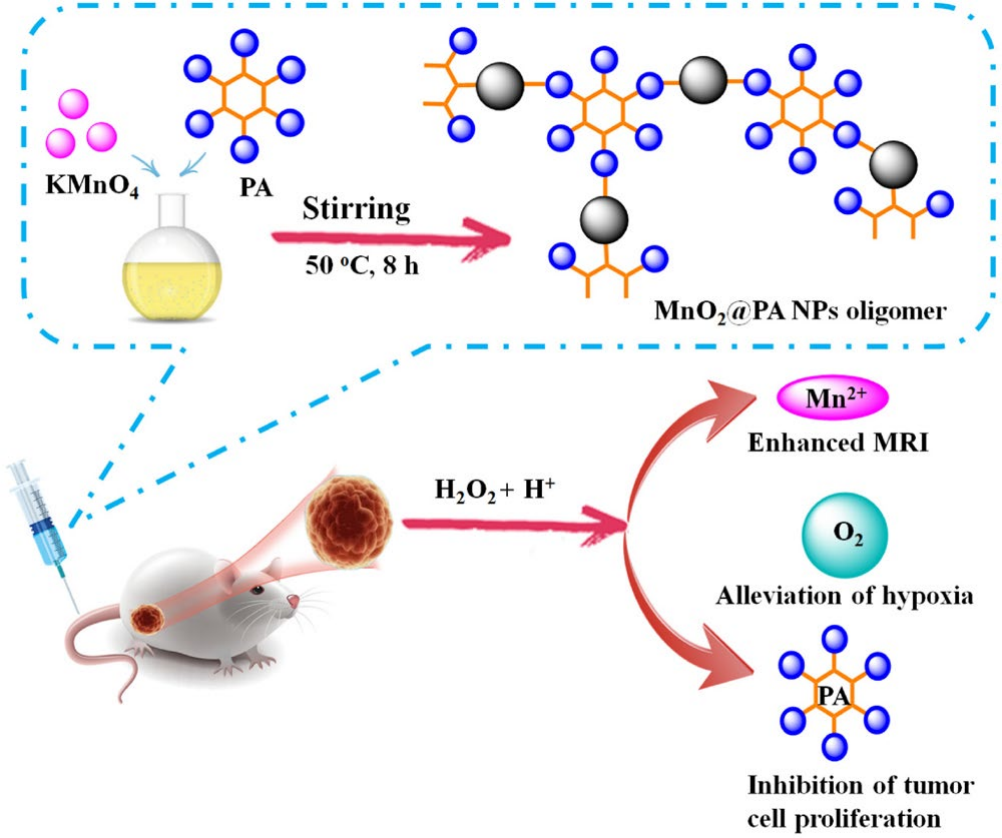
Fabrication of MnO_2_@PA NPs oligomer for *in vivo* MRI and therapy of osteosarcoma.

**Figure 2.**
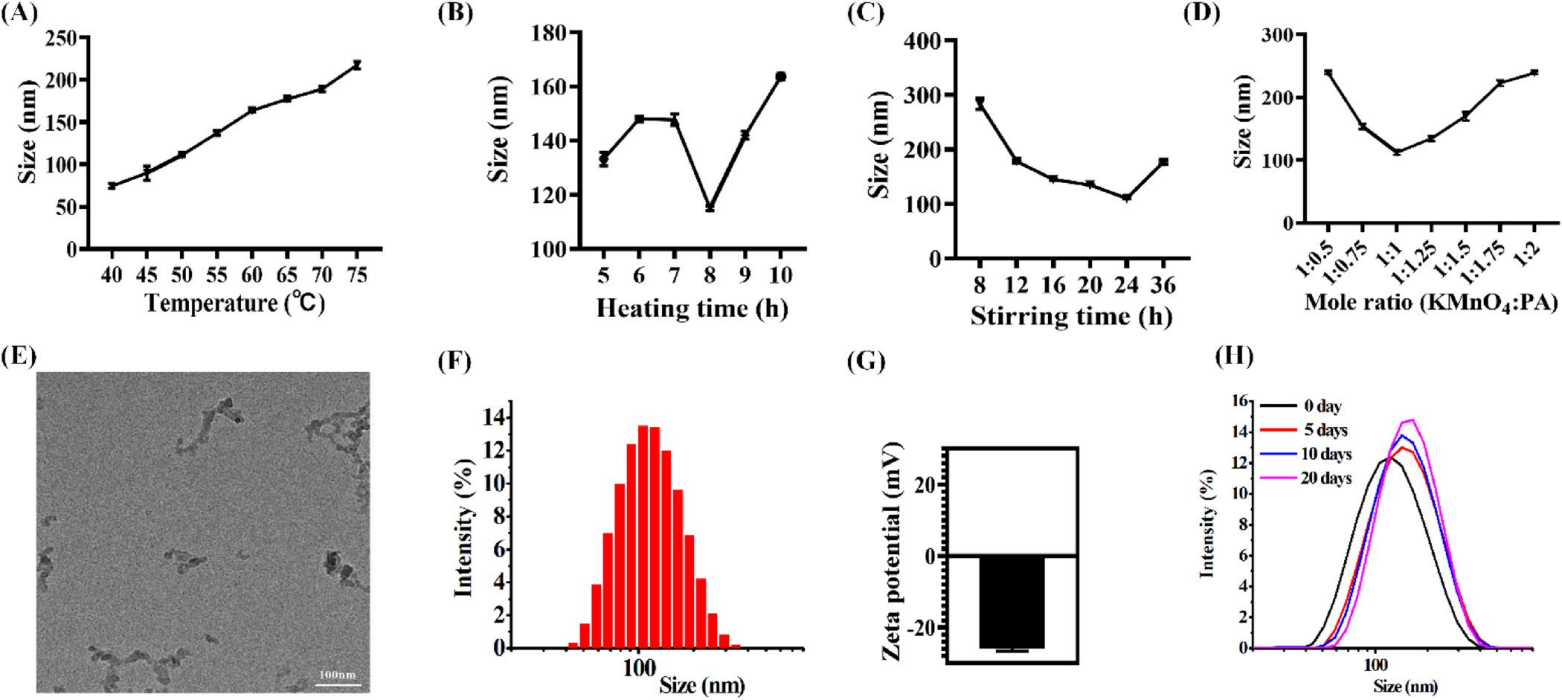
A–D: The size distribution of MnO_2_@PA NPs oligomer under various preparation conditions. (A) Reaction temperature, (B) heating time, (C) stirring time, and (D) various molar ratio of KMnO_4_ and PA. E: The morphology of MnO_2_@PA NPs oligomer observed by TEM, (Scale bar: 100 nm). F and G: The size distribution and zeta potential of NPs. H: The size distribution of MnO_2_@ PA NPs oligomer in water under various storage time.

Reduction of KMnO_4_ is a classical method to prepare MnO_2_ NPs (Wang et al., 2017; Gao et al., 2021). Otherwise, MnO_2_ NPs can be modified through addition of various ligand (Ma et al., 2021; Zhang et al., 2020) or cell membranes (Huang et al., 2022). MnO_2_ NPs or its derivatives were widely used for tumor treatment because MnO_2_ can react with H_2_O_2_ in tumor region to produce O_2_ to alleviate tumor hypoxia (Lin and Zhao et al., 2018). PA is a natural extracts from plant seeds and widely used as additives, antioxidant, and chelating agent (Bloot et al., 2021). Interestingly, recent research demonstrated that PA exhibited antitumor activities and special bone targeting ability (Zhou et al., 2019; Wang et al., 2020). Based on these results, we speculated that PA-modified MnO_2_ NPs can enhance the antitumor activity of MnO_2_ and can serve as targeting therapeutics for osteosarcoma treatment. To verify the hypothesis, the PA-modified MnO_2_ NPs was prepared through a simple method (Figure 1). The synthesis method was optimized by adjusting reaction temperature, time, as well as reactant ratio. As shown in Figure 2(A), the size of MnO_2_@PA NPs was obviously increased with higher reaction temperature (Figure 2A). Interestingly, with prolonged heating time from 5 h to 6 h, the size of NPs increased obviously. However, when heating time was increased to 8 h, the size of NPs decreased dramatically. Otherwise, the size of NPs increased again when prolonging the heating time continuously (Figure 2(B)). Longer stirring time was also beneficial to decreasing the size of NPs. However, when prolonging the stirring time to 36 h, the size of NPs increased obviously (Figure 2(C)). The size of NPs was also significantly affected by the ratio of KMnO_4_ and PA. As displayed in Figure 2(D), when the ratio of KMnO_4_ and PA was 1:1, the size of NPs reached the minimum.

The morphology of NPs was confirmed by TEM. Interestingly, TEM results indicated that MnO_2_@PA NPs oligomer was formed (Figure 2(E)). The reason may be that the six phosphate of PA can bond several manganese, which caused the MnO_2_@PA NPs oligomer formation. In addition, DLS results demonstrated that the size of MnO_2_@PA NPs oligomer was 111.1 ± 1.9 nm (Figure 2(F)) and the PDI was 0.28 ± 0.03. Otherwise, the zeta potential of MnO_2_@PA NPs oligomer was –25.9 ± 0.6 mV (Figure 2(G)). Interestingly, the size of MnO_2_@PA NPs oligomer was not significantly increased when stored in water for 20 days (Figure 2(H)). These results indicated that the MnO_2_@PA NPs oligomer showed good stability in water.

**Figure 3.**
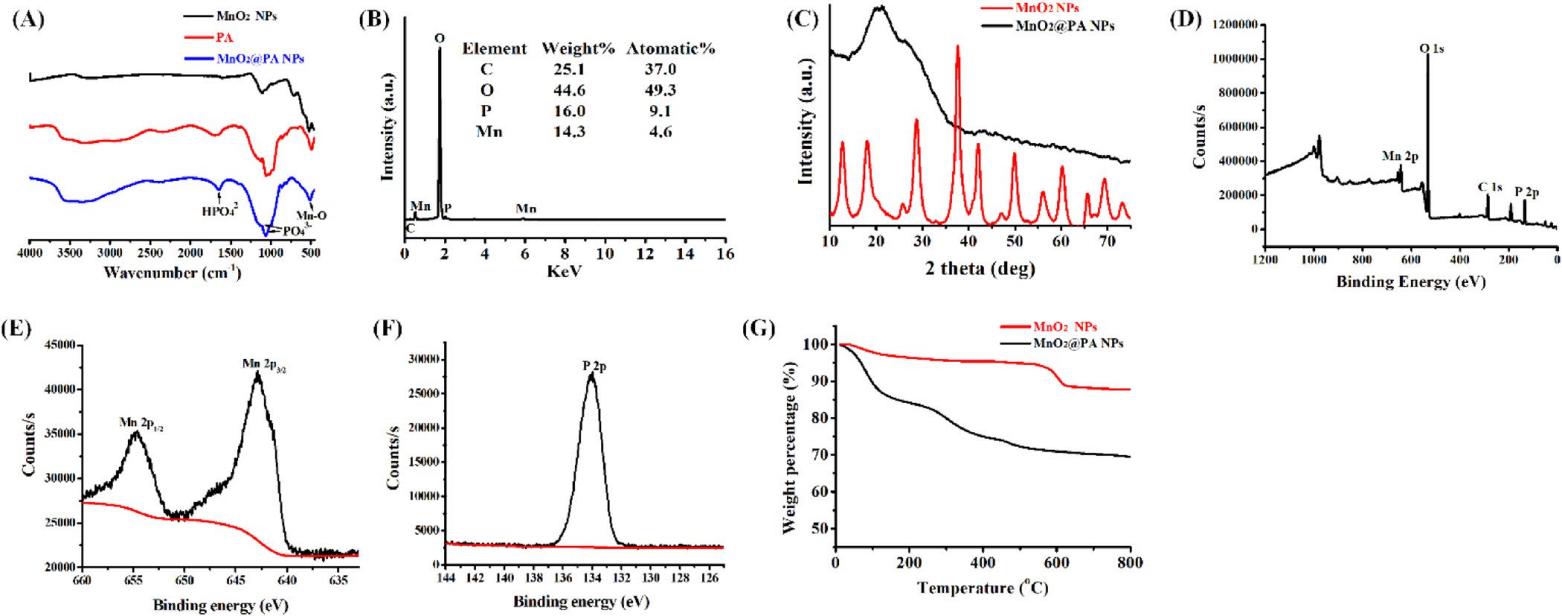
Characterization of MnO_2_@PA NPs oligomer. A: FTIR spectra of PA, MnO_2_ NPs, and MnO_2_@PA NPs oligomer. B: EDS spectra of MnO_2_@PA NPs oligomer. C: XRD spectra of MnO_2_ NPs and MnO_2_@PA NPs oligomer. D–F: XPS spectra of MnO_2_@PA NPs oligomer, Mn and P elements. G: The thermogravimetric curves of MnO_2_ NPs and MnO_2_@PA NPs oligomer.

The composition of MnO_2_@PA NPs oligomer was confirmed by FTIR and EDS analysis. The FTIR spectra of MnO_2_@PA NPs oligomer showed that Mn-O stretching vibration was observed at 514 cm^−1^ (Figure 3(A)). In addition, the absorption peak of HPO_4_^2–^ and PO_4_^3–^ appeared in the absorption spectrum of MnO_2_@PA NPs (Figure 3(A)), indicating that PA was successfully modified on MnO_2_@PA. The EDS analysis results showed that C, Mn, O, and P elements was observed in MnO_2_@PA NPs (Figure 3(B)), this result further demonstrated that MnO_2_@PA NPs was successfully modified by PA.

The XRD diffractograms of MnO_2_ NPs showed many diffraction peaks, which indicated that the MnO_2_ NPs had a highly crystalline structure (Figure 3(C)). However, amorphous structure was found in the MnO_2_@PA NPs, which implied that the crystal morphology of MnO_2_ was destroyed by PA modification. The XPS results showed that the signals of Mn, O, C, and P could be observed in MnO_2_@PA NPs, this results further demonstrated that PA was successfully modified on MnO_2_ NPs (Figure 3(D)). Otherwise, the spin-orbit coupling level splitting peaks of Mn2p_3/2_ and Mn2p_1/2_ appeared at 642 eV and 655 eV (Figure 3(E)), and the spectral peak of P2p appeared at 134 eV (Figure 3(F)), respectively. These data further indicated that the MnO_2_@PA NPs were successfully fabricated and the structure of MnO_2_@PA NPs was amorphous. PA may change the surface free energy of the MnO_2_ crystal, which may influence the growth rate of the crystal surface according to the Curie–Wulifu principle. Consequently, the heterogeneous crystal surface may disrupt the crystal morphology of MnO_2_. In addition, we used α-Al_2_O_3_ as a control to analyze the thermal stability of the synthesized NPs under nitrogen atmosphere. As shown in the Figure 3(G), 15% and 2% weight loss was observed in MnO_2_@PA NPs and MnO_2_ NPs in the low temperature range, respectively. The weight loss may be attributed to the loss of water and the absorbed PA. MnO_2_@PA NPs lose 15.6% weight between 150 and 600 °C, while MnO_2_ NPs only lost 5.2% weight during the same temperature range (Figure 3(G)). The difference of thermogravimetric loss of the NPs may be contributed to the decomposition of PA in MnO_2_@PA NPs. All the results confirmed that MnO_2_@ PA NPs oligomer has been successfully fabricated and its structure is amorphous.

### 3.2. Cytotoxicity assay

**Figure 4.**
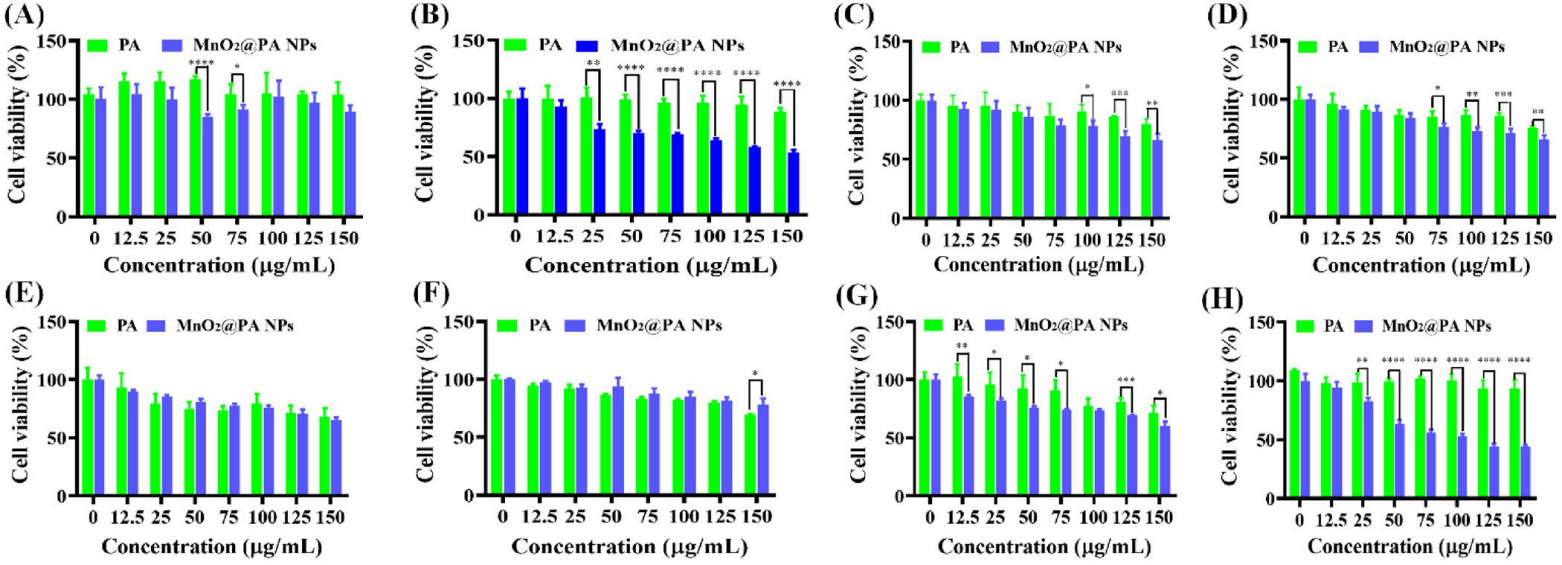
Cytotoxicity of PA and MnO_2_@ PA NPs oligomer on macrophages (A), U2OS cells (B), 4T1 cells (C), Ishikawa cells (D), MC 38 cells (E), Panc 02 cells (F), SW480 cells (G), and 143B cells (H) were measured by CCK-8 assay with 4-h incubation. The data were shown as mean ± SD. *p < .05, **p < .01, ***p < .001, and ****p < .0001 vs. PA group.

The cytotoxicity of PA and MnO_2_@PA NPs in RAW 264.7 cells and tumor cells were evaluated by CCK-8. As shown in Figure 4(A), both PA and MnO_2_@PA NPs displayed poor cytotoxicity on RAW 264.7 cells with 4-h incubation under various concentration. The cell viability of 4T1, Ishikawa, MC 38, Panc 02, and SW480 cells were decreased with high concentration of PA treatment after 4-h incubation (Figure 4(C–G)), suggesting that PA can inhibit these tumor cells proliferation. In addition, PA displayed poor cytotoxicity on U2OS and 143B cells (Figure 4(B,H)). Interestingly, the cell viability of all the tested tumor cells were decreased with high concentration of MnO_2_@PA NPs treatment (Figure 4(B–H)). Importantly, compared to PA, the cell viability of U2OS, 4T1, Ishikawa, SW480, and 143B were significantly decreased with high concentration of MnO_2_@PA NPs treatment (Figure 4(B–D,G,H)). These results implied that MnO_2_@PA NPs exhibited enhanced *in vitro* antitumor activity compared to PA.

After 24-h incubation, the cell viability of RAW 264.7 cells was not significantly decreased with high concentration of PA treatment (Figure S1A, Supporting Information). However, the cell viability of U2OS, 4T1, MC 38, Panc 02, and SW480 cells were obviously decreased with high concentration of PA treatment after 24-h incubation (Figure S1(B,C,E–G), Supporting Information). These results indicate that the antitumor activity of PA was enhanced on U2OS, 4T1, MC 38, Panc 02, and SW480 cells with prolonged incubation time. Interestingly, the cell viability of U2OS, 4T1, Ishikawa, Panc 02, and 143B cells were distinctly decreased by MnO_2_@PA NPs treatment compared to that of PA after 24-h incubation (Figure S1(B–D,F,H), Supporting Information). These results further demonstrated that MnO_2_@PA NPs displayed increased antitumor activity compared to PA.

### 3.3. Cellular uptake

**Figure 5.**
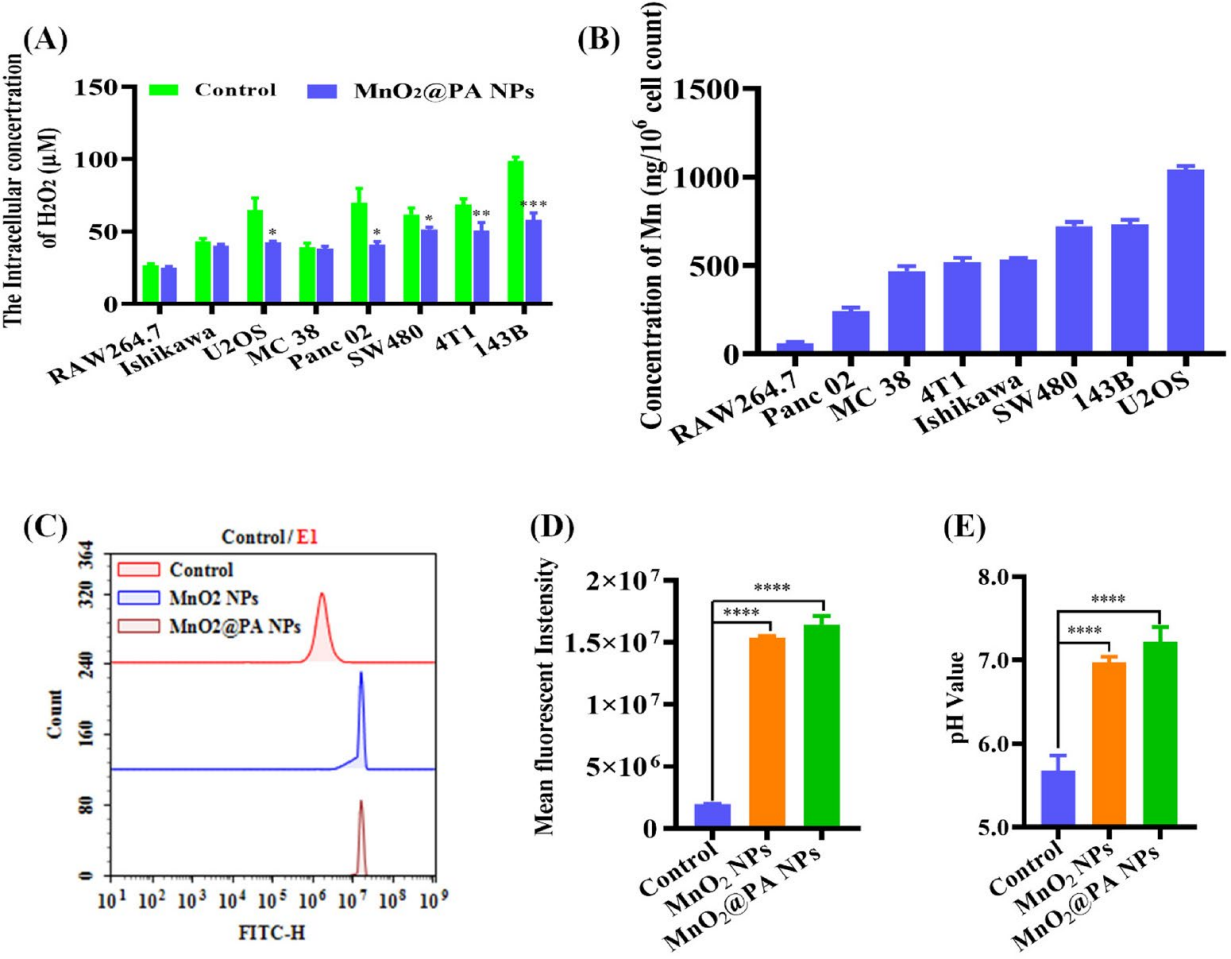
A: Detection of intracellular hydrogen peroxide concentrations after MnO_2_@PA NPs oligomer treatment. B: The cellular manganese contents when cells treated with MnO_2_@PA NPs oligomer. C and D: The fluorescence intensity of cells when cells treated by MnO_2_ NPs or MnO_2_@PA NPs oligomer (Strong fluorescence indicating to weak acid conditions). E: The intracellular pH values when cells treated by MnO_2_ NPs or MnO_2_@PA NPs oligomer. The data were shown as mean ± SD. *p < .05, **p < .01, ***p < .001, and****p < .0001 vs. control group.

To investigate whether MnO_2_@PA NPs can be internalized by macrophage or tumor cells, the manganese contents in cells were determined by ICP-MS. As shown in Figure 5(B), only a small amount of manganese were detected in RAW 264.7 cells, suggesting that only few MnO_2_@PA NPs was internalized by macrophages. Interestingly, the manganese contents in MC 38, 4T1, and Ishikawa is close to 500 ng per 10^6^ cells, indicating that MnO_2_@PA NPs can be efficiently internalized by these cells. Furthermore, the manganese contents in SW480 and 143B cells were further increased, indicating that the cellular uptake of MnO_2_@PA NPs was enhanced by these cells. It is worth noting that the highest manganese contents were detected in U2OS cells, implying that U2OS cells had the strongest cellular uptake ability for MnO_2_@PA NPs compared to other tumor cells.

### 3.4. Detection of ROS and H_2_O_2_ concentrations in cells

NPs can significantly influence the ROS concentration in cells (Sun et al., 2022). Otherwise, MnO_2_ NPs can react with ROS such as H_2_O_2_ to produce O_2_ to alleviate tumor hypoxia (Guo et al., 2020). Therefore, the cellular ROS concentrations were detected with MnO_2_@PA NPs treatment. As shown in Figure 5(A), the fluorescence intensity of tumor cells was obviously increased when cells were treated by MnO_2_@PA NPs compared to that of negative control. Semi-quantitative analysis results were consistent with the fluorescence intensity determination (Figure S2, Supporting Information). These results demonstrated that the uptake of MnO_2_@PA NPs can elevate the intracellular ROS concentrations, which is consistent with literature report (Hafez et al., 2019). The reason may be that MnO_2_ NPs only reacted with H_2_O_2_ to produce O_2_ (Jiang et al., 2020), which didn’t alter other ROS such as •OH, O_2_^–^, and ^1^O_2_.

To further investigate the influence of MnO_2_@PA NPs on ROS concentration varieties, the intra- and extracellular H_2_O_2_ concentration in RAW 264.7 and tumor cells were also detected. When cells treated with MnO_2_@PA NPs, the extracellular H_2_O_2_ concentration of all cells was not changed obviously (Figure S3, Supporting Information). In contrast, compared to control group, the intracellular H_2_O_2_ concentration of U2OS, 4T1, Panc 02, SW480, and 143B was significantly decreased. As previously described, MnO_2_@PA NPs can be efficiently internalized by tumor cells, and the internalized NPs can react with H_2_O_2_ to produce O_2_, which may reduce the intracellular H_2_O_2_ concentration.

As mentioned, MnO_2_ NPs can react with H_2_O_2_ under acid condition to produce O_2_. Therefore, the pH value viability in cells was detected. As shown in Figure 5(C–E), the pH value in 143B cells was obviously increased when cells treated with MnO_2_ or MnO_2_@PA NPs compared to that of untreated group. MnO_2_ can exhaust H^+^ to react with H_2_O_2_ to produce O_2_, consequently, the cellular pH values were increased when cells were treated with MnO_2_ or MnO_2_@PA NPs. In addition, MnO_2_@PA NPs treated cells has higher cellular pH value than that of MnO_2_ group. The reason could be that MnO_2_@PA NPs can be easily internalized by tumor cells than MnO_2_ NPs. All the results demonstrated that the internalized MnO_2_@PA NPs can react with H_2_O_2_ under acid conditions to produce O_2_ to alleviate tumor hypoxia.

### 3.5. In vivo imaging on a 143B tumor-bearing mice model

**Figure 6.**
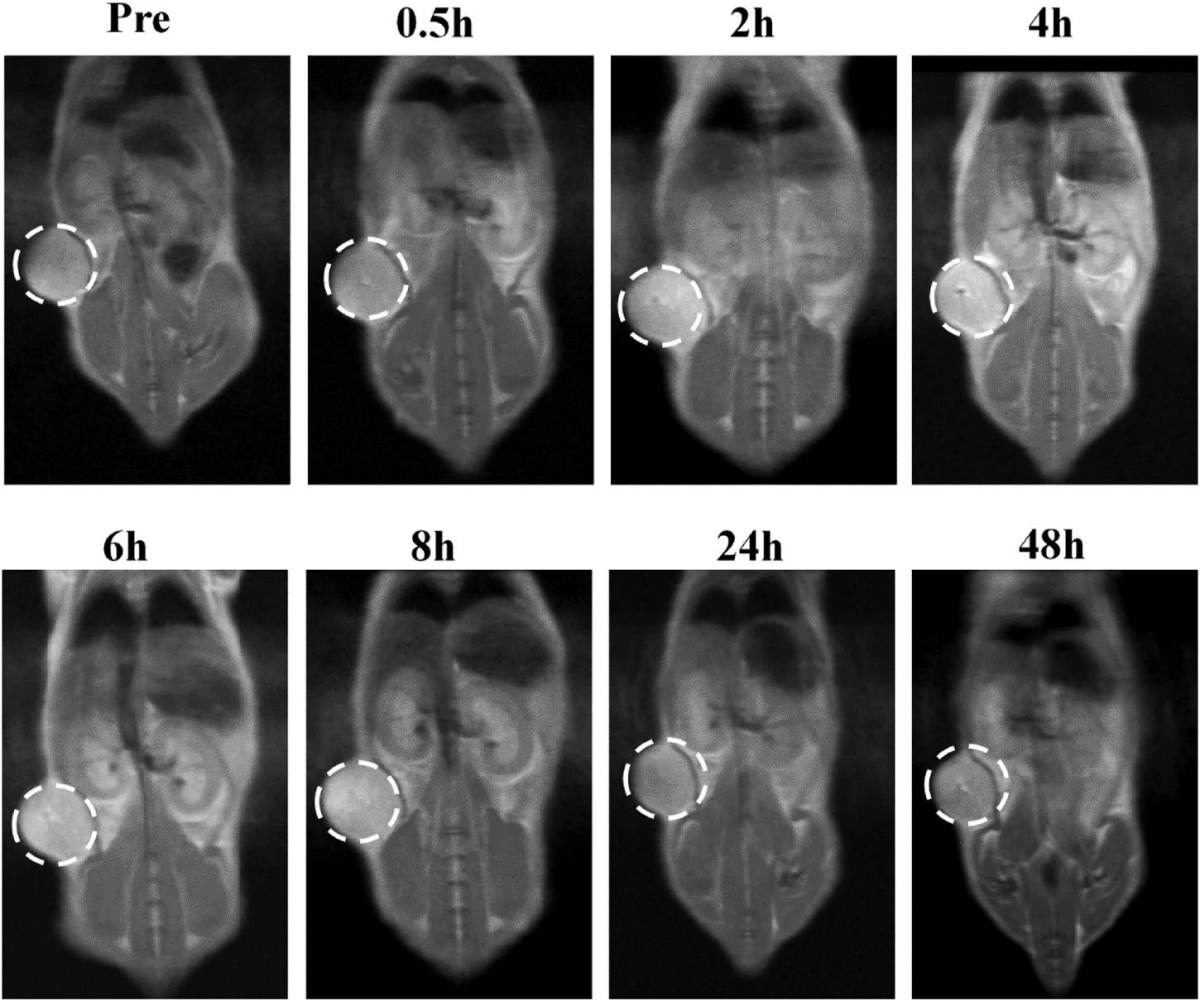
*In vivo* T1-weighted MRI of 143B tumor-bearing mice after 0.5, 2, 4, 6, 8, 24, and 48 h injection of MnO_2_@PA NPs oligomer.

MnO_2_ NPs can react with H_2_O_2_ to produce Mn^2+^, which can serve as T1-weighted MRI contrast for tumor (Lin et al., 2018). Consequently, we checked the T1-weighted MRI ability of MnO_2_@PA NPs in tumor-bearing mice. After injection of 20 mg/kg NPs, the T1-weighted MRI at 0.5, 2, 4, 6, 8, 24, and 48 h were recorded. As shown in Figure 6, no enhanced MRI were observed after 0.5 h injection. Interestingly, an enhanced MRI was obtained when mice received MnO_2_@PA NPs after 4, 6, and 8 h injection. In addition, the MR signals were attenuated after 24 or 48 h injection. These results indicated that MnO_2_ NPs can be passively accumulated in tumor tissues and the accumulated NPs can produce Mn^2+^ under acidic tumor microenvironment. To further investigate if MnO_2_@PA NPs can be cleaned from body, the T1-weighted MRI for kidney in tumor-bearing mice was performed. As shown in Figure S4 (Supporting Information), an enhanced T1 signal was observed in the kidney after 4 h injection, and the MR signal was obviously attenuated after 24 h injection, suggesting that the MnO_2_@PA NPs can be cleaned from body by kidney.

### 3.6. In vivo antitumor efficacy

**Figure 7.**
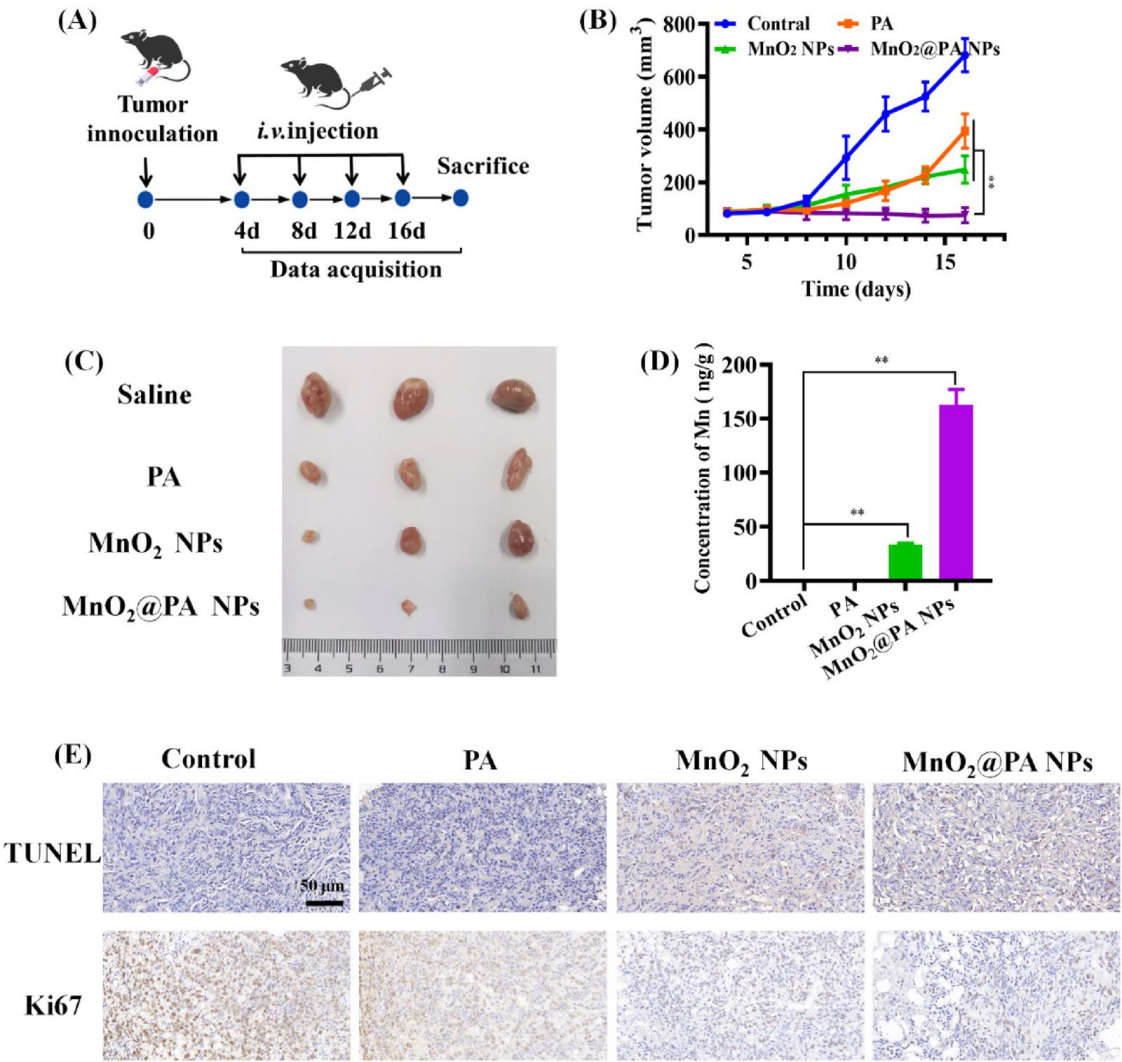
*In vivo* antitumor efficacy evaluation of PA, MnO_2_ NPs and MnO_2_@PA NPs oligomer in 143B tumor-bearing mice. A: The dosage frequency of therapeutics after tumor inoculation. Mice received 20 mg/kg of PA, MnO_2_ NPs or MnO_2_@PA NPs. B: The tumor growth curves after i.v. injection of PA, MnO_2_ NPs, and MnO_2_@PA NPs oligomer. The data were shown as mean ± SD, **p < .01 vs. PA group and MnO_2_ NPs group. C: The excised tumor images with PA, MnO_2_ NPs, or MnO_2_@PA NPs oligomer treatment. D: The contents of manganese in excised tumor. The data were shown as mean ± SD, **p < .01 vs. control group. E: TUNEL detection and immunohistochemistry assay for Ki67 in tumor tissues, (scale bar: 50 μm).

*In vitro* experiments demonstrated that MnO_2_@PA NPs can significantly decrease tumor cells viability, which inspired us to further investigate the *in vivo* antitumor ability. The *in vivo* antitumor efficacy of the NPs was evaluated using 143B tumor-bearing mouse model. As shown in Figure S6 (Supporting Information), all of the tumor-bearing mice maintained their weight during the treatment. PA and MnO_2_ NPs could obviously inhibit tumor growth compared to saline group during the treatment (*Figure 7(B)*), because PA and MnO_2_ NPs had been confirmed owning antitumor activity *in vivo* (Masunaga et al., 2019; Liu et al., 2022). Importantly, tumor growth was significantly inhibited when mice treated with MnO_2_@PA NPs compared to that of PA and MnO_2_ NPs after 16 days of treatment (Figure 7(C)), indicating that MnO_2_@PA NPs treatment had better antitumor activity than PA or MnO_2_ NPs. The reason may that MnO_2_@PA NPs can combine the antitumor activity of PA and MnO_2_ NPs (Figure 7(D)), which cause MnO_2_@PA NPs show better *in vivo* antitumor activity than other groups. In addition, we also detected the apoptosis of cancer cells after therapeutics treatment by TDT-mediated dUTP nick end labeling (TUNEL) assay (Figure 7(E)). Compared with saline group, MnO_2_@PA NPs could significantly induce 143B cell apoptosis, which was consistent with *in vivo* antitumor results. In summary, MnO_2_@PA NPs showed enhanced *in vivo* antitumor activity compared to PA or MnO_2_ NPs.

### 3.7. Preliminary evaluation of the biosafety of MnO_2_@ PA NPs

Healthy Kunming mice were selected to evaluate the biosafety of MnO_2_@PA NPs. There was no significant difference in body weight and organ weight when mice treated with different concentrations of MnO_2_@PA NPs or saline (Figure S8(A,B), Supporting Information). Otherwise, the hematological parameters of mice treated with MnO_2_@PA NPs did not show significant difference compared to saline group (Figure S7, Supporting Information). Typical biomarker related to hepatic and renal functions such as alanine aminotransferase (ALT), aspartate aminotransferase (AST), creatinine (CREA), and urea also did not show obvious difference compared to saline group. H&E staining showed no changes in heart, liver, spleen, lung, kidney, necrosis, congestion, or vascular morphology (Figure S8(C), Supporting Information). These results demonstrate that MnO_2_@PA NPs has good biocompatibility and no obvious adverse effects when administrated intravenously.

## 4. Conclusion

Patients with osteosarcoma remain high mortality after surgery. Herein, we developed a PA-modified MnO_2_ NPs by a simple method for targeting MRI and therapy of osteosarcoma. The physicochemical properties of MnO_2_@PA NPs were thoroughly characterized by FTIR, XRD, XPS, EDS, TEM, and TGA. The results demonstrated that PA was successfully modified on MnO_2_ NPs, and the structure of MnO_2_@PA NPs is amorphous. TEM results verified that a MnO_2_ oligomer was formed by PA modification. The MnO_2_@PA NPs oligomer has uniform size distribution and negative zeta potential. *In vitro* experiments confirmed that MnO_2_@PA NPs oligomer could be internalized by various tumor cells, and the internalized MnO_2_ could react with H_2_O_2_ to produce Mn^2+^ and O_2_ under acid microenvironment. *In vivo* experiments demonstrated that MnO_2_@PA NPs oligomer can accumulate in tumor tissues, and the accumulated NPs can release Mn^2+^ and O_2_ for T1-weighted MRI and targeting therapy of osteosarcoma, respectively. Compared to MnO_2_ NPs and PA, MnO_2_@PA NPs oligomer can significantly inhibit tumor growth without obvious adverse effects. Our results provide novel candidate for targeting MRI and therapy of osteosarcoma.

## Supplementary Material

Supplemental Material
