# Supplementary material for: Phytic acid-modified manganese dioxide nanoparticles oligomer for magnetic resonance imaging and targeting therapy of osteosarcoma
Source: Drug Deliv. 2023 Mar 1;30(1):2181743. doi: 10.1080/10717544.2023.2181743 (PMC9980014; doi:10.1080/10717544.2023.2181743)
Supplement: Supplemental Material [file IDRD_A_2181743_SM6325.docx]

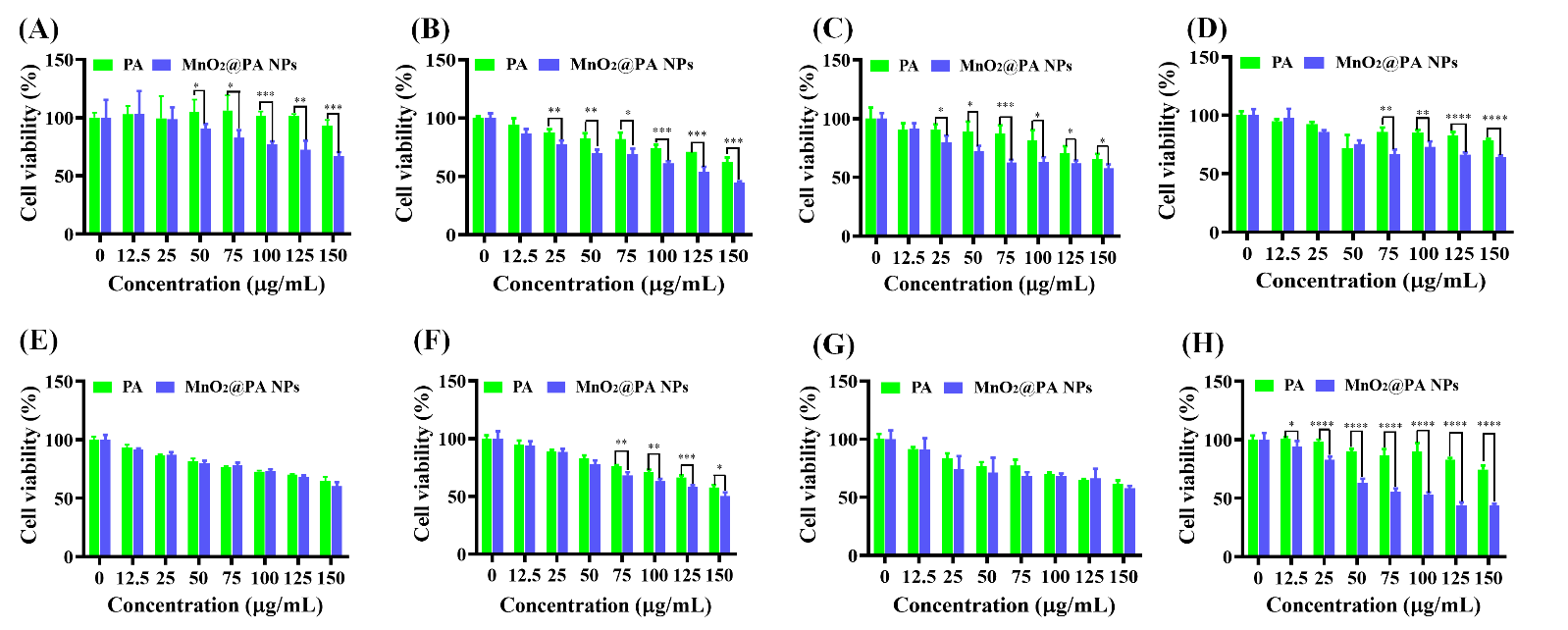


Figure S1. Cytotoxicity of PA and MnO_2_@ PA NPs oligomer on macrophages (A), U2OS cells (B), 4T1 cells (C), Ishikawa cells (D), MC 38 cells (E), Panc 02 cells (F), SW480 cells (G) and 143D cells (H) were measured by CCK-8 assay after 24 h incubation. The data were shown as mean ± SD. *p < 0.05, **p <0 .01, ***p <0 .001 and ****p < 0 .0001 vs. PA group.


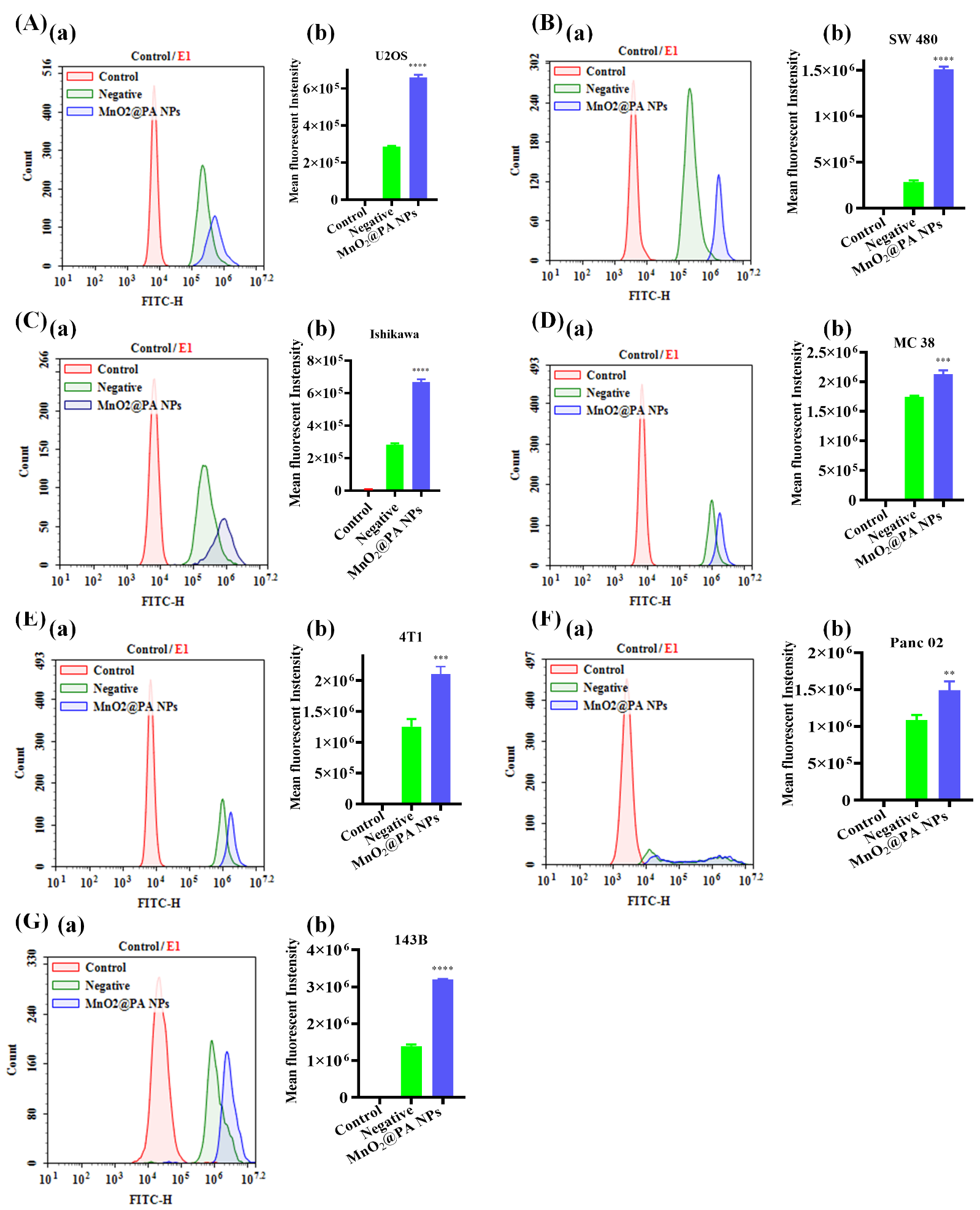


Figure S2. Intracellular ROS was detected by flow cytometry in U2OS cells (A), 4T1 cells (B), Ishikawa cells (C), MC 38 cells (D), Panc 02 cells (E) and SW480 cells (F), and 143B cells (G). (a) Fluorescence intensity indicating ROS concentration; (b) The quantitative analysis of fluorescence intensity. The data were shown as mean ± SD. ***p < 0 .001 and ****p < 0 .0001 vs. control group.

Figure S3. Detection of extracellular hydrogen peroxide concentrations after MnO_2_@PA NPs oligomer treatment. The data were shown as mean ± SD. *p < 0 .05 vs. control group.


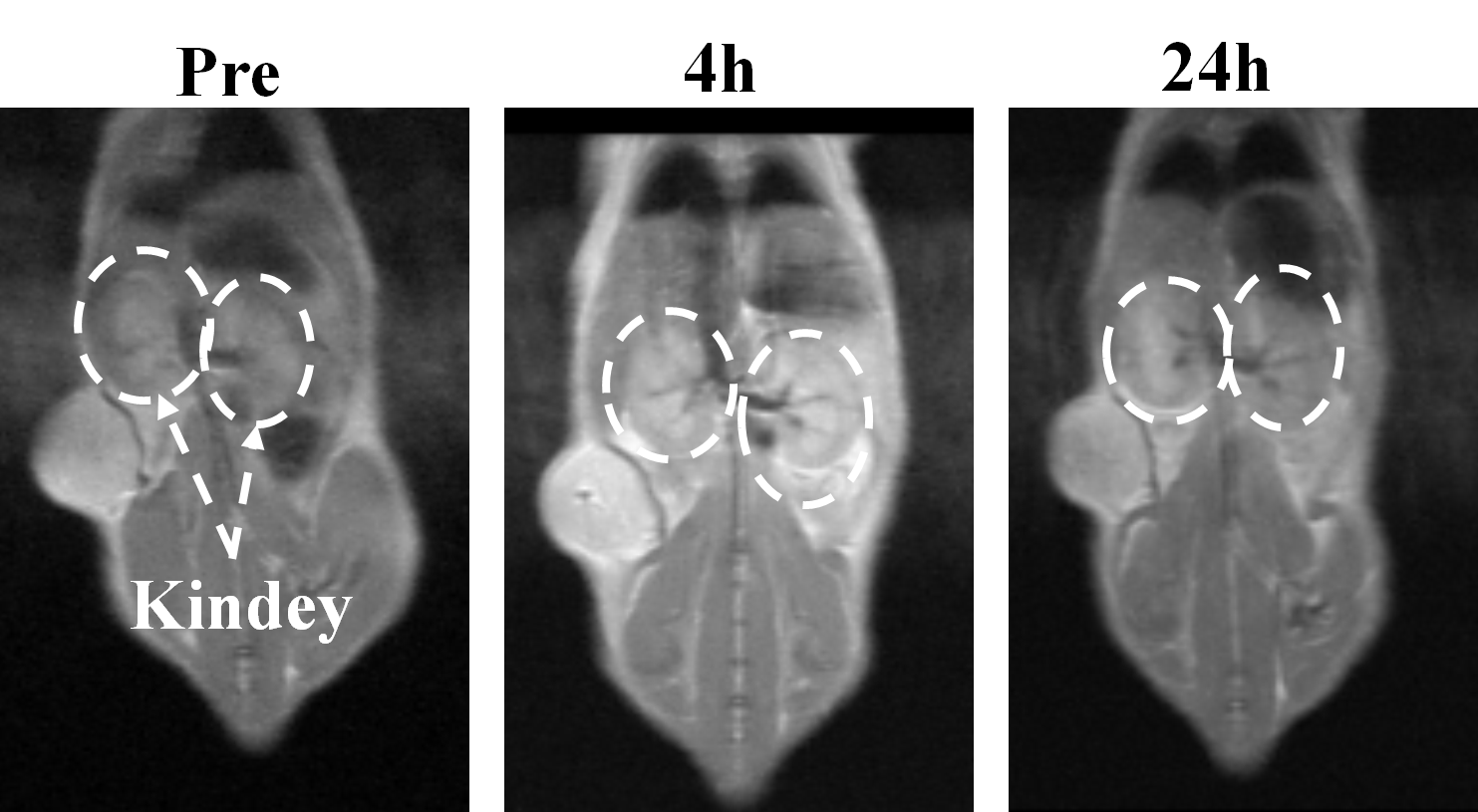


Figure S4. *In vivo* T1-weighted MRI of 143B tumor-bearing mice after 4 h and 24 h injection of MnO_2_@PA NPs oligomer.





Figure S5. T1-weighted MRI of tumor-bearing mice after with various therapeutics treatment.

Figure S6. Body weight of tumor-bearing mice during treatment.


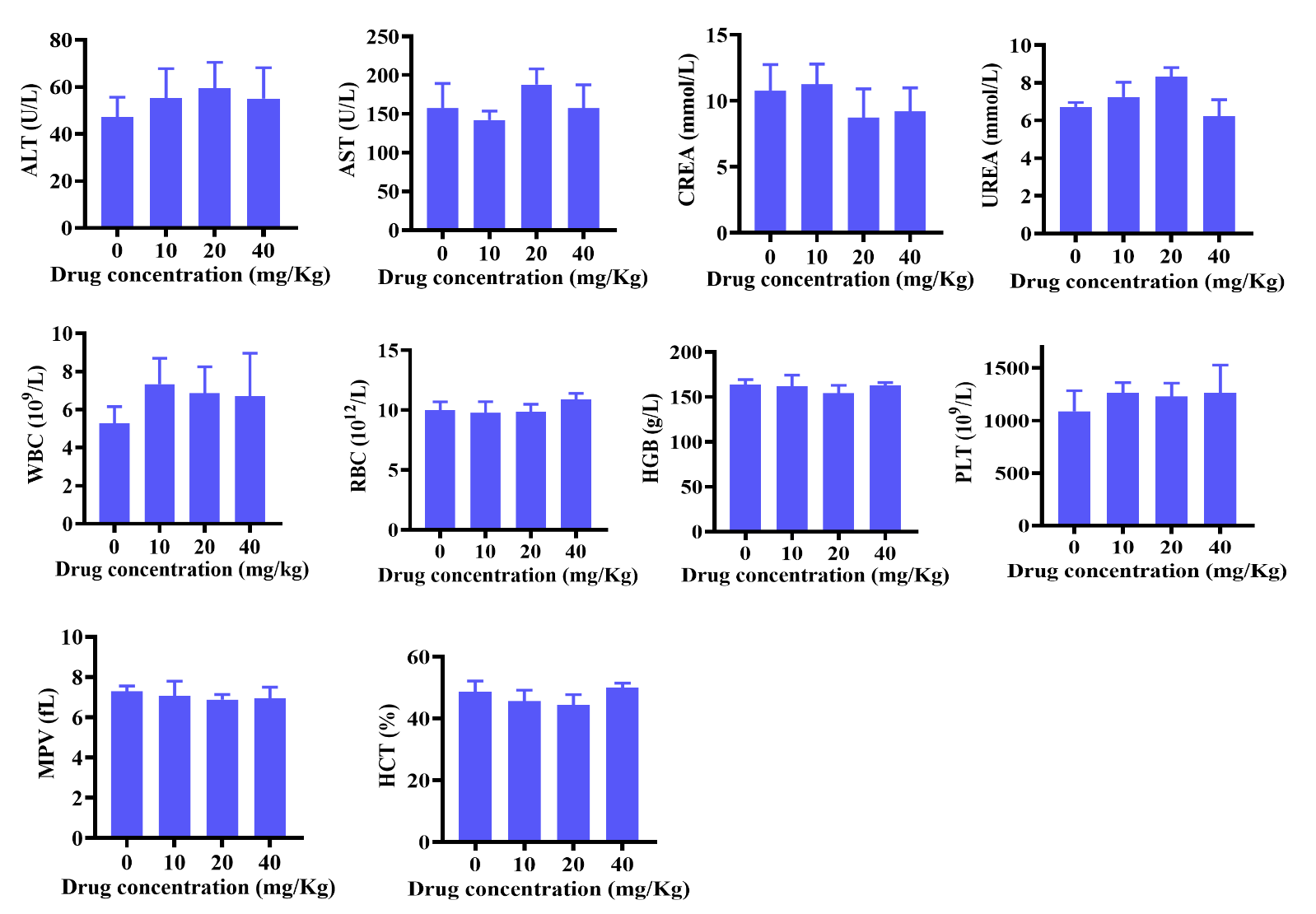


Figure S7. Hematological parameters, liver and kidney function indexes of mice treated with various concentration of MnO_2_@PA NPs oligomer.


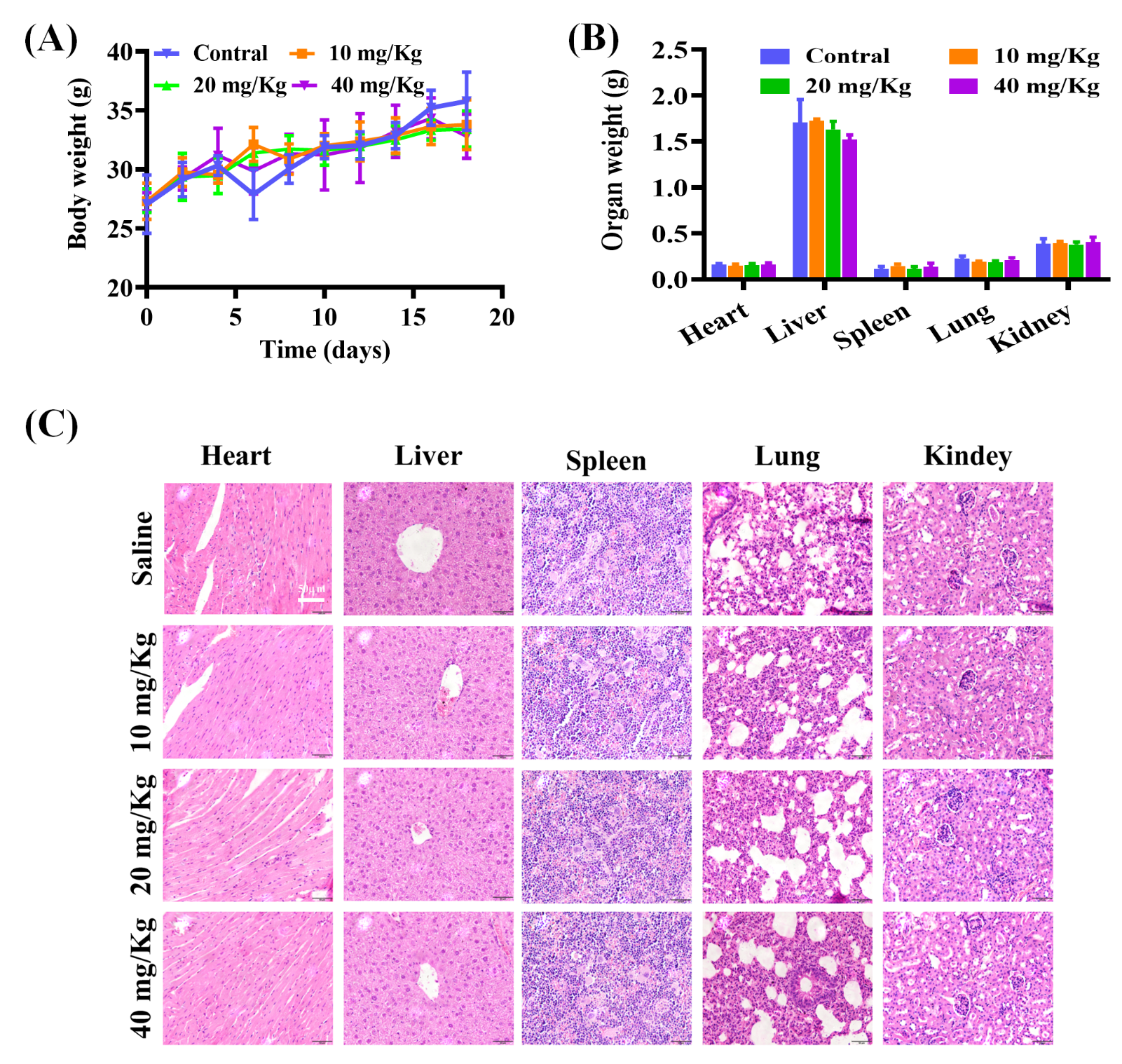


Figure S8. Mice body weight (A) and organ weight (B) after 16 days of MnO_2_@PA NPs oligomer treatment. (C) H&E staining of main organs of mice, (scale bar: 50 μm).
